# An original Arduino-controlled anaerobic bioreactor packed with biochar as a porous filter media

**DOI:** 10.1016/j.mex.2021.101615

**Published:** 2022-01-06

**Authors:** Yusuf Küçükağa, Andrea Facchin, Cristian Torri, Serdar Kara

**Affiliations:** a Department of Chemistry “Giacomo Ciamician”, University of Bologna, via Sant'Alberto, 163, 48123, Ravenna, Italy; b Environmental Engineering Department, Faculty of Engineering, Gebze Technical University, 41400, Kocaeli, Turkey

**Keywords:** MMC, Mixed microbial culture, COD, Chemical Oxygen Demand, O_2_, Oxygen Gas, PA12, Polyamide, OD, Outer Diameter, ID, Inner Diameter, V:V, Volume to Volume ratio, M:M, Mass to Mass ratio, LOD, Limit of Detection, H_2_, Hydrogen Gas, GC, Gas Chromatography, TCD, Thermal Capture Detector, MS, Mass Spectrometer, Arduino digital controller, Single-board microcontroller, Digital gasometer, 3D printing, Biochar filter, Mixed microbial culture, Anaerobic fermentation, Lab-scale fermenter, Custom-made reactor, Inexpensive bioreactor system

## Abstract

Bioreactors are commonly used apparatuses generally equipped with several built-in specifications for the investigation of biological treatment studies. Each bioreactor test may require different types of specialty such as heating, agitation, re-circulation and some further technologies like online sensoring. Even thought, there are many ready-to-use fabricated bioreactors available in the market with a cost usually over than 1000 €, it is often not possible to access those advanced (but inflexible) systems for many students, young-researchers or small-scale private R&D companies. In this work, a new low cost (≈100€) packed-bed anaerobic bioreactor was developed, and all methodological details including open-source coding and 3D design files are shared with informative descriptions. Some preliminary tests were conducted to verify the developed bioreactor system's credibility in terms of leak-tightness, accurate gas monitoring, temperature controlling, and mass balance (COD-eq) coverage, which all have shown a very promising performance.

• A consistent model bioreactor that will be called as “tetrapod” was developed for anaerobic treatment of challenging substrates such as pyrolytic liquids.
• Coarse biochar grains were used as an organic packing material to stimulate the microbial bioconversion by increasing the active surface area for the attached-growth anaerobic mixed microbial culture (MMC).
• An open-source Arduino based digital gasometer was developed for online monitoring of biogas change in the lab-scale system. Arduino was also used as a digital controller for maintaining pulse-mode liquid recirculation of the bioreactor.

## Graphical abstract

**Figure:**
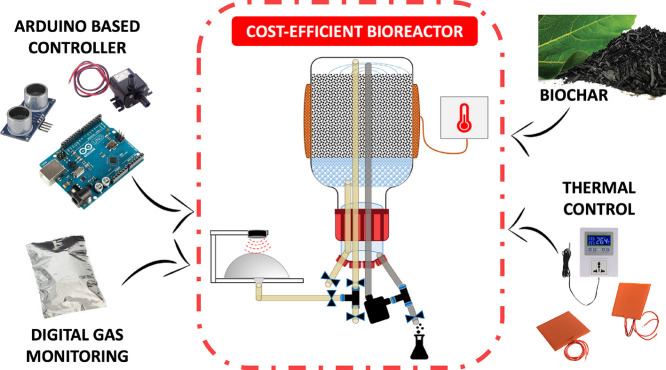

Specifications table

**Table:** 

Subject Area:	Environmental Science
More specific subject area:	Anaerobic Biotechnology
Method name:	A cost-effective Arduino supported biochar-packed bioreactor system developed for anaerobic bio-utilization of unconventional substrates
Name and reference of original method:	Not applicable
Resource availability:	All can be found in Additional Information with all the details including the cost-estimation for the developed bioreactor system.

## Method details

### Background

Anaerobic biotechnological methods have been drawing great attention with an increasing importance [1]. Anaerobes do not only allow biological treatment of highly polluted waste-streams, yet it creates another possibility to re-valorize the input materials by bioconversion into several end-products such as biogas, bio-fuels, commodity chemicals and so on [2]. Packed-bed or trickling-bed bioreactors (TBRs) are one of the most preferable, easy-to-built and well-known tools for conducting anaerobic biological processes, where the anaerobic microorganisms are aimed to be fixed to a packed filter media rather than being suspended in a slurry [3]. This way of biofilm-based bioreactor technologies mainly provides a higher conversion yield because of a better interaction occurring between substrate material and the microbes [4]. This also provides a less-suspended, much clearer effluent as compared to slurry systems [5]. Sands, stones, ceramics and recently some polymeric materials have been commonly used as packing bed media [4,6]. However, there is a little attempt to use non-inert materials such as pyrolysis derived biochar, which have a great potential for stimulating biological activities with its unique porous structure [7], [8], [9], [10], [11].

The use of biochar in biological systems has recently started to be seen as a very promising and efficient strategy for enhancing the anaerobic microbial activity [12,13]. Some of the main advantages of the application of charcoal-like pyrolysis derived biochar material, for enhanced anaerobic digestion by means of its unique physiochemical porous structure, are reported as; contribution to pH buffering [14], mitigation of several inhibition phenomenons such as ammonia and volatile fatty acids (VFA) inhibitions [15], [16], [17], providing a faster start-up period by shortening the lag-phase duration [7], stimulating the substrate removal rate resulting in a better product (i.e. biogas) yield [18]. Although there are several promising reports about the use of biochar for anaerobic digestion purposes (i.e. biomethanation) are available in the literature, no attempt was found for the use of biochar material as a packing material for the more specific anaerobic biotechnology purposes such as acidogenic fermentation (VFA production), solventogenic bioreactions (e.g. bio-ethanol synthesis), anaerobic conversion of gaseous materials, or bio-utilization of unconventional substrates (e.g. aqueous pyrolytic liquid).

Single-board microcontrollers are known as easy-to-use and affordable digital controller systems. Arduino is one of the most widely known microcontrollers which is based on open-source Integrated Developing Environment (IDE). With its cost-competitive hardware tools and free-to-access software, Arduino can also serve as a very useful platform for digital controlling of special-made reactors for biochemical research purposes. The cost-benefits make this microprocessor widely affordable, even for quite complicated tasks such as the control of a lab-scale bioreactor. The main use of Arduino in chemistry is the field of automated bioreactor monitoring and datalogging (also known as “ChemDuino”), whereas is less frequently applied for bioreactors controlling [19,20].

In order to investigate the anaerobic conversion of unconventional substrates (e.g., pyrolytic liquids, syngas) flexible, robust and easily customizable bioreactors are needed. That kind of bioreactors often require controlling of some operational parameters such as liquid/gas flow, temperature, and agitation etc. Besides, a real-time monitoring of the bio-chemical reactions (e.g., biogas production rate, pH monitoring etc.) are also substantial in most of the cases. This work targeted two main aspects which are relevant for that purpose:

1. Packed-bed bioreactor assembly that allow to obtain satisfactory mass and chemical oxygen demand (COD) balance, suitable for the study of different coarse filling materials.
2. Simple real time monitoring system for reactor controlling and gas production.

In this study, an Arduino-based digital-controlling system was developed to control the lab-scale anaerobic bioreactor which is going to be also called as ‘tetrapod’. Moreover, an original digital gasometer with a 3D-Printed holding structure was developed by using an Arduino-based electronic system, for easy and inexpensive way of monitoring the online gas amounts derived from the conducted bioreactions in the bioreactor system.

### Design and construction of the bioreactor

**Fig. 1:**
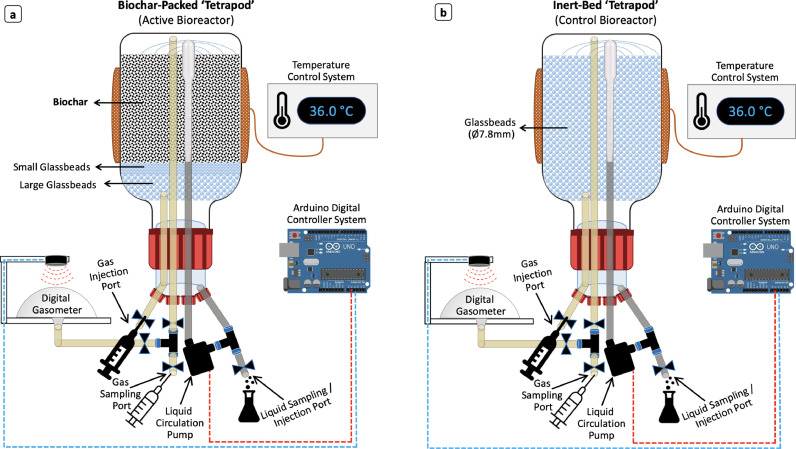
Schematic diagram of the experimental equipment: (a) Biochar-packed tetrapod, (b) Inert-bed tetrapod.

Schematic diagram of the tetrapod bioreactor system is presented in Fig. 1. Shortly, the bioreactor system consists of three parts:

1. Glass bottle part that is equipped with the heating system: A standard half-liter laboratory type of glass-bottle (i.e., duran bottle, pyrex glass-bottle) was used upside-down.
2. Four-ported special-cap together with liquid recirculation pump: There are four ports available at the bottom for the purposes of gas injection, gas sampling, liquid sampling/injection, and liquid recirculation. This peculiar cap was the inspiration of the ‘tetrapod’ naming due to its appearance. The glass-mouth, the four screw-openings and the surrounding screw-caps were manufactured as water-tight materials by using silicone O-ring septa for each screw cap. Nonetheless, since bio-liquid fills the cap from inside, this avoids the risk of any gas leakage from the bottom. In this way undissolved gas molecules would stay in the top head-space which is the bottom of the glass-bottle where there is no leaking risk.
3. Digital gasometer system for biogas storage and quantification: The change in produced and consumed gas amounts in the system was monitored online with an Arduino based digital gasometer which was developed by the research team. Detailed description of the digital gasometer will be explained under the relevant sub-section.

Two identical tetrapod bioreactors were assembled, but with different packing materials (Fig. 4) . First one with the coarse biochar packing material which will be called ‘biochar-packed tetrapod’ or active bioreactor. The latter is ‘inert-bed tetrapod’ (also control bioreactor) where the glassbeads were the only packing material. The control bioreactor with inert-bed was designed to evaluate the effect of biochar addition in terms of anaerobic bioconversion efficiency. Biochar used as packing material is a commercial charcoal provided by “Romagna Carbone s.n.c.” (Italy) which was obtained from orchard pruning biomass (apple, grapevine, pear, peach) with a slow pyrolysis process at 500 °C and a residence time of 3 h, in a kiln of 2.2 m in diameter and holding around 2 tons of feedstock [21].

In anaerobic cultivation reactors, especially when involving the strict-anaerobes, it is very important to guarantee a perfect sealing all through the system to avoid any cross-contamination of gasses. There are two main reasons for this; one is that anaerobic microbes are quite sensitive to oxygen exposure, and the second is to obtain a good mass and COD balance of the system. Preliminary tests showed that the main experimentally relevant issue of small-scale reactor is the air leakage, namely the input of air into the reactor without significant change into gas volume. Without special devices and using standard approaches for anaerobic digestion tests, a COD loss mainly (but probably not only) due to oxygen (O_2_) permeation into silicon hoses, was found and estimated in almost 38 mL-*d*^−1^ of oxygen. Such phenomena, even if acceptable for short term anaerobic digestion of biodegradable substrates, is not acceptable for long term studies of challenging substrates, in which long lag phases could increase the length of the study and, consequentially, the total COD loss. To fix the O_2_ permeation issue, several mock tetrapod bioreactors structures were assembled, filled with hydrogen or helium (most penetrable gasses) and tested for gas leaks through gas analysis of the headspace through time. Such tests provided some key hints to minimize leakages.

**Fig. 2:**
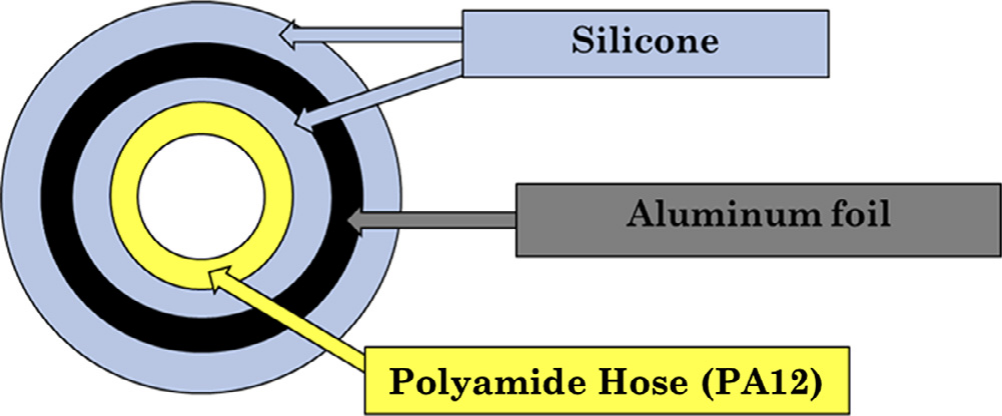
A cross-section drawing of the modified pneumatic hoses of the ‘tetrapod’ bioreactor.

First the ‘tetrapod’ bioreactor was designed to have all joints and sealing submerged in water. This allows to easily detect any leaky joints and, thanks to low gas solubility in the liquid, to minimize permeation of gasses through correctly sealed joints. Besides joints, the external hoses, connectors, and sampling valves revealed to be a large source of minor leaks due to gas permeation and relatively high surface area (given small reactor volume), and therefore were improved to obtain a perfectly gas-tight system. For this purpose:

• All the external equipment was connected by quick-connect pneumatic connectors which are well-used in gas-tight systems for industrial purposes. All the pneumatic connection adapters and the sampling valves showed a good barrier performance.
• Tubing was performed with laminated hoses, which were manufactured by wrapping an aluminum foil onto polyamide hoses (PA12) using silicone as layer binder and final coating (Fig. 2).

**Liquid recirculation and sampling:** It is an important operational procedure to ensure the homogeneity of packed-column bioreactor and to keep the bio-filter media wet, which is a critical issue for the attached-growth microbes. Moreover, in order to allow the contact between biofilm and gasses the packed-column pores should not be permanently filled with liquid. Given the variable hydraulic conductivity of packed column, overflowing of bioliquid above the filter media is also another critical point to be avoided. For this reason, a regulatable pulsed mode of water recirculation system was applied for the ‘tetrapod’ bioreactor. An Arduino based script allows the submersible-type mini pump to work for adjustable time, with a flow capacity of 240 L/hr. Therefore, circumventing the somewhat difficult procurement of a pump with specific discharge rate, which in turn depends on the hydraulic conductivity of the filling media. Digital controlling code of the water recirculation system and electrical connection schema of the pump are provided in detail at the Supplementary Material together with a further explanation and current market prices of each component used.

The mini pump was connected to a hose through the same multilayer approach. First the hose was fitted to the pump, thereafter a silicone layer was applied to seal the two parts. To prevent any acetic acid contamination, usually presented in silicone paste, the system was left working with water overnight. Then, all the acetate-contaminated washing-water was discharged.

The sprinkling system at the top of filter bed was built using a modified plastic Pasteur pipette (Fig. 1). Pipette filler was used as a terminal part of the recirculation system. The opposite part (tip) was firstly cut to remove the narrowing needle part, then connected to a hose and fixed with a cable tie. On the pipette filler, identical (≈ 2.0 mm) holes were made with a hot soldering needle in a symmetric distribution that allows a homogenous liquid distribution to all directions. The size of the holes was tuned in order to force the liquid to spread onto entire packed bed and to avoid clogging with entrained particles.

Bio-liquid samplings were made by using disposable plastic syringes via ‘liquid sampling / injection port’ equipped with a quick-connect pneumatic valve. The liquid port was placed just before the water-pump for sustaining a well-mixed representative liquid sampling.

**Digital gasometer and gas sampling:** To provide a real-time monitoring of gas amount in the tetrapod system, a “digital gasometer” was designed and manufactured. Briefly, the device uses the distance between the ultrasonic sensor and the top of a flexible gas-bag (which is inversely proportional to the amount of gas in the bag) to determine the total available gas volume in the gas-bag. Gas-bag expands while filled with gas, reducing the distance between the top layer of it and the sensor.

**Fig. 3:**
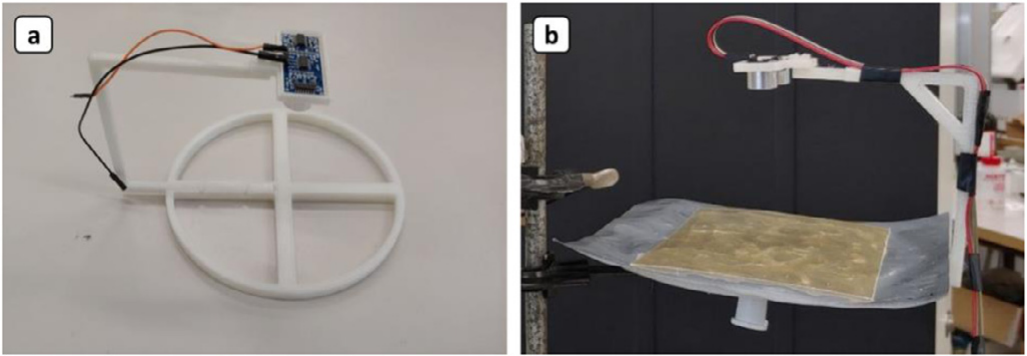
(a) 3D-Printed PLA structure assembled with HC-SR04™ Ultrasonic sensor. (b) Final structure of the Digital Gasometer with a Supel™ 30,226-U gasbag and a rectangular plastic piece (yellow) to homogenize the surface (right).

The custom-made digital gasometer system consists of an HC-SR04™ ultrasonic sensor, an Arduino board, and a Supel™ 30,226-U gasbag with 1 liter capacity who was fixed to a 3D-Printed holding structure developed to perfectly fit the sensor and the gas-bag. 3D design files (.stl) and Arduino sketches downloadable from the Supplementary Material. The back side of gasbag was faced the ultrasonic sensor (Fig. 3). To improve the ultrasound reflecting behavior, a rectangular rigid plastic piece was glued on the top of the gas-bag (Fig. 3, b).

Gas samplings throughout the experiments were made using a quick-connect valve of the ‘gas sampling port’ by using graduated disposable syringes which allow withdrawal of a known amount of gas sample. Prior to each gas sampling, the headspace gas and the gasometer gas was mixed by using the sampling syringe. Such operation allowed a better homogenization between the gasbag and the headspace of the reactor, providing a much representative gas sample from the bioreactor system.

**Arduino digital controller:** An Arduino Mega microcontroller was used to control the digital gasometer and the liquid recirculation. In this method, Arduino was used to monitor digital gasometer and control the liquid recirculation. Ultrasonic sensor on the digital gasometer continuously reads and send distance values to Arduino. Then, the microcontroller directly converts the distance values into volume data through an external software (such as “*Programino software*”), record and save the reading data on a “.txt” file. The developed Arduino sketch provides a fixed time between each reading of volume, thus knowing the initial time, it's possible to monitor the time of the gas volume changes.

**Temperature control system:** Heating and temperature control system were designed to operate the bioreactor at the desired mesophilic temperature range (34–38 °C). The heating system consists of two elastic resistance pads placed on the wall of the bioreactor bottle, a thermocouple fixed to the liquid recirculation hose, and a digital thermostat that provides power to the resistances depending on the measured temperature value. The digital thermostat had a 0.1 °C level of sensitivity for adjusting the set temperature level. Each component of the heating system and its electrical circuit diagram is described in the Additional Information. Moreover, a folding thermal jacket (made by aluminum foil) was placed to cover the entire walls of the glass bottle for sustaining a constant and homogeneous distribution of temperature conditions in the packed-bed reactor (Fig. 5c).

### Experimental set-up

**Fig. 4:**
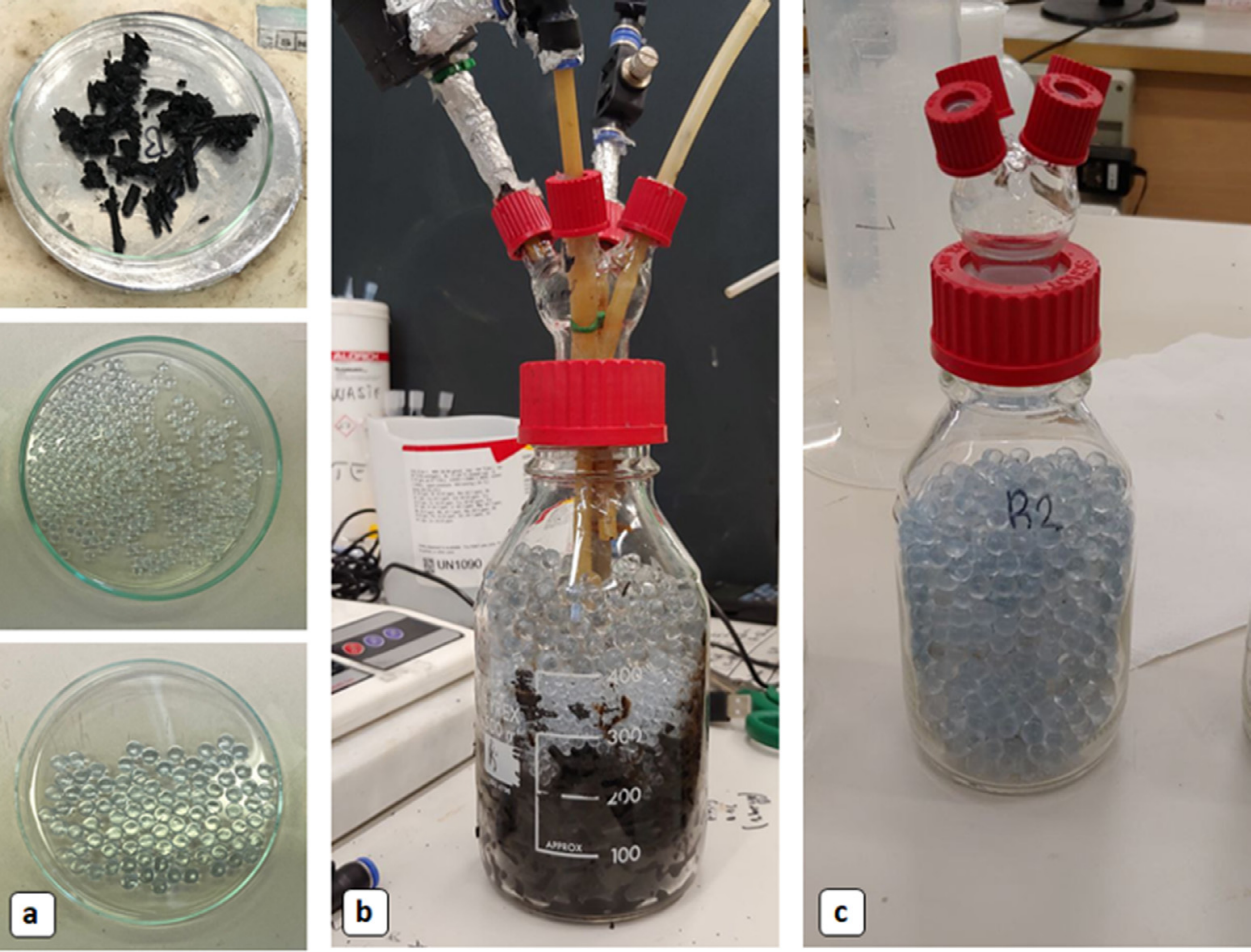
(a) Packing materials; wetted coarse biochar grains (top), small glassbeads with Ø4.0 mm (middle), large glassbeads with Ø7.8 mm (bottom). (b) Biochar amended tetrapod supported with small beads and large beads. (c) Inert-bed tetrapod filled only with large glassbeads as packing material.

Two identical tetrapod bioreactors were filled with different packing materials, as mentioned before (Fig. 4). Table 1 is providing all set-up details of the assembled tetrapod bioreactors. The inert-bed control reactor was filled with 480 g (dry-weight) of large glassbeads with 8 mm outer diameter (OD) (Fig 4.a bottom), which corresponds to ≈305 (± 15) mL net filter-bed volume in total. The active bioreactor was packed with coarse biochar grains, which was supported onto two layers of different size of glassbeads. To prevent biochar grains entraining into recirculation flow, 325 g of small glassbeads with 4 mm OD were layered below biochar and, 200 g of large glassbeads (OD 8 mm), below them. The multilayer glassbead media as a retainer for the upper biochar-media part, was corresponding around 205 mL net occupied volume and the rest filter-bed volume was occupied by coarse biochar grains.

**Table 1:** Set-up parameters of the bioreactors.

Parameters / Details	Biochar-Packed ‘Tetrapod’ (Active Bioreactor)	Inert-Bed ‘Tetrapod’ (Control Bioreactor)
Reactor Type	Anaerobic Packed-Bed Bioreactor	Anaerobic Packed-Bed Bioreactor
Filter Type	Carbonous Porous Media	Non-porous Inert Media
Packing Material	Coarse Biochar Grains + Small and Large Glassbeads	Large Glassbeads
Operating Temperature	36 °C ± 2	36 °C ± 2
Liquid Recirculation Pump Capacity	220 L/hr	220 L/hr
Reactor Total Empty Volume	620 mL	620 mL
Total Net Filter-Bed Volume	≈ 305 ± 15 mL	≈ 305 ± 15 mL
Filter Media Ratio (V:V)	Biochar Grains: Small Beads: Large Beads * 36%: 40%: 24%	Large Beads 100%
Total Liquid Volume	200 mL	200 mL
Headspace Volume	≈ 115 ± 15 mL	≈ 115 ± 15 mL
Total Biochar Amount	100 g (in wet basis⁎⁎)	n/a

⁎ Grain diameters of the glassbead filter medias were; Ø7.8 mm for large ones, and Ø4.0 mm for small ones.

⁎⁎ Biochar water content was 69% (M:M).

Assembling of tetrapods was made following a restricted procedure that allows to set-up the bioreactor in the best configuration possible for gas and liquid recirculation. Firstly, keeping the bottle vertical, the gasbag tube connector and liquid recirculation tube with the modified Pasteur pipette, were inserted in the central part of the half-liter sized bottle. Thereafter, biochar grains, small glassbeads, and big glassbeads were added for the biochar packed tetrapod. For the control reactor all big glassbeads were added in one time. The addition of all layers allowed to fix the long tubes previously inserted. Tubes had to be longer than the bottle itself, so that after the connection of the special cap, there should be a residual outside part for connecting all the necessary external equipment (pump and digital gasometer). After this step, the specially-designed screw cap was sealed to the bottle. The special cap was designed with four small ports (6 mm ID and 18 mm OD) on the external side and with a restricted neck in the inside part. This restriction allowed to avoid falling down of filter media grains (glassbeads) into the internal side of the four-small ports. The last two tubes of the tetrapods, for liquid output and gas injection port were connected at the end. The gas input tube should enter some millimeters inside the big glassbeads layer if possible. The liquid output tube, instead, must enter as short as possible inside the cap, in order to avoid dead volumes. Finally, the caps were completely sealed, and the pump was connected to the recirculation system. Before to connect the digital gasometer, assembled tetrapod must firmly be turned upside down, allowing the inside layer to maintain the exact order needed. After the bioreactor was fixed into a stable support at upside down position, the digital gasometer and the heating system were attached.

**Fig. 5:**
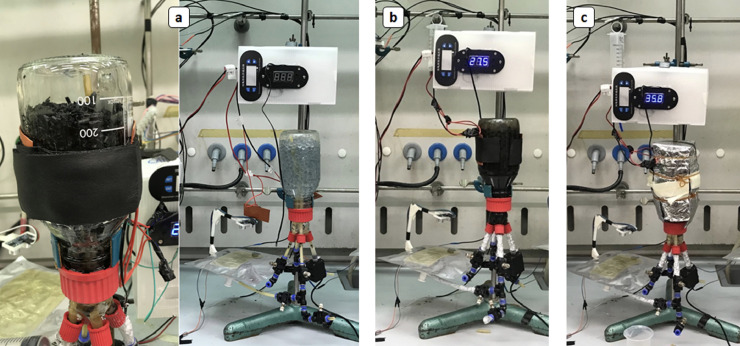
Operational start-up steps of the ‘Tetrapod’ bioreactor system: **(a)** Assembled tetrapods before operation; biochar packed bioreactor on the left, inert-bed bioreactor on the right. **(b)** Freshly inoculated tetrapod with anaerobic MMC. **(c)** An on-going experiment of one tetrapod covered with a thermal jacket.

Pictures were taken from the start-up of the first anaerobic experiment conducted in the assembled tetrapod system. Fig. 5a shows the clean biochar-packed bioreactor on the left and clean inert-bed bioreactor on the right, prior to the operation. In Fig. 5b, one of the tetrapods was recently inoculated and started an anaerobic test. After the start-up, the tetrapod was covered by the thermal-jacket to keep the bioreactor in a more standardized temperature condition (Fig. 5c).

## Method validation

### Digital gasometer calibration and test

**Fig. 6:**
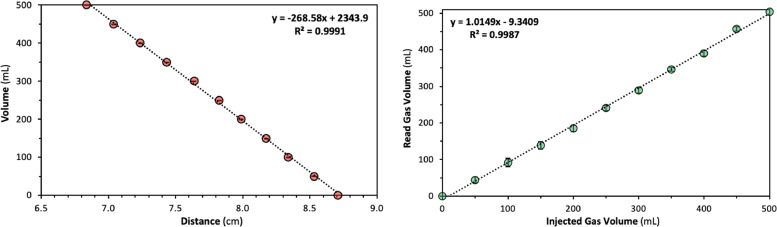
(a) Calibration graph of the Digital Gasometer. (b) Accuracy test of the Digital Gasometer.

The digital gasometers were calibrated several times to sustain a linear correlation between distance and gas volume. A 100 mL graduated syringe was connected to the gasbag via a three way-valve for gas charging and discharging cycles of calibration. The calibration was conducted with 50 mL intervals by sequential gas charging up to 500 mL. After it reached the total volume of 500 mL, it was discharged with 50 mL intervals again until all the gas was totally removed. In each interval point, corresponding distance read by the ultrasonic sensor of the Arduino was recorded. Prior to the ultimate calibration a minor difference was noticed in the corresponding interval points of charging and discharging cycles, due to a possible small difference in the gasbag precise position. Such intervention was initially minimized by fixing the gasbag to the 3D-Printed support using silicone as a glue and the calibration was continued. The explained calibration procedure was repeated three times and the distance values were averaged, which should provide a linear correlation between distance and gas volume. In Fig. 6a calibration curve of the digital gasometer is shown. From the calibration data, the digital gasometer revealed an average sensitivity of ≈25 mL/mm. The accuracy of the system was evaluated by introducing known amounts of volume in the instrument, results are shown in Fig. 6b. The average accuracy error was calculated as 5%, with a limit of detection (LOD) of 9 mL.

**Fig. 7:**
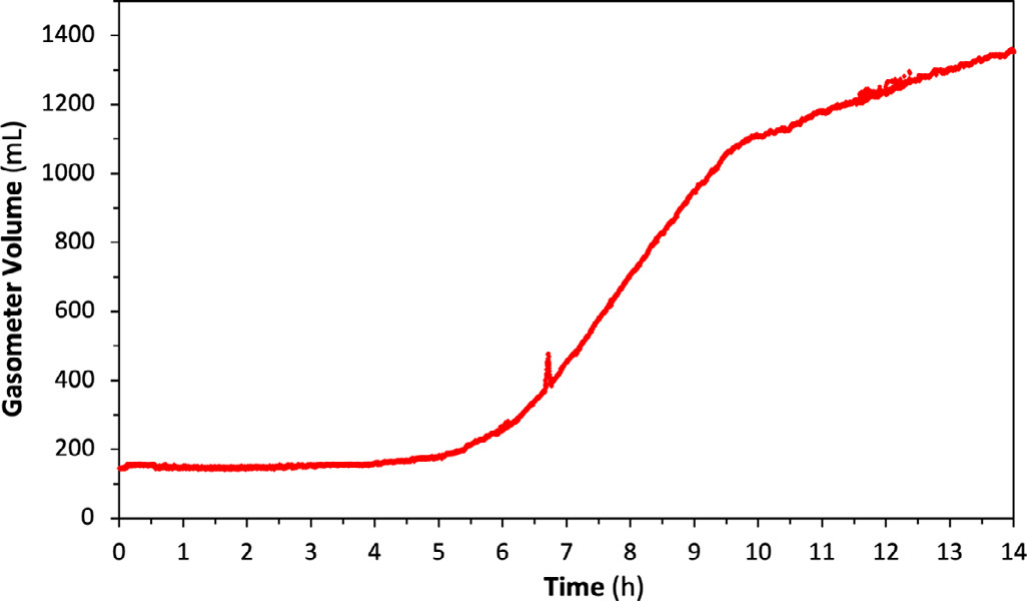
Biogas production from MMC fermentation of glucose.

The correlation was then uploaded into the Arduino script, allowing to display the current available gas volume. The digital gasometer and Arduino script was tested within a real biogas production test. For this purpose, one tetrapod bioreactor was inoculated with a digestate sample obtained from a local commercial anaerobic digester treating grape pomace and wastewater treatment sludges. Produced biogas amounts was monitored by the calibrated digital gasometer overnight. Total duration of the biogas production test was 14 hr and the digital gasometer conducted each reading every 5 s. Fig. 7 shows the biogas production trend of the conducted test, which was obtained by the digital gasometer.

### Temperature controlling efficiency

**Fig. 8:**
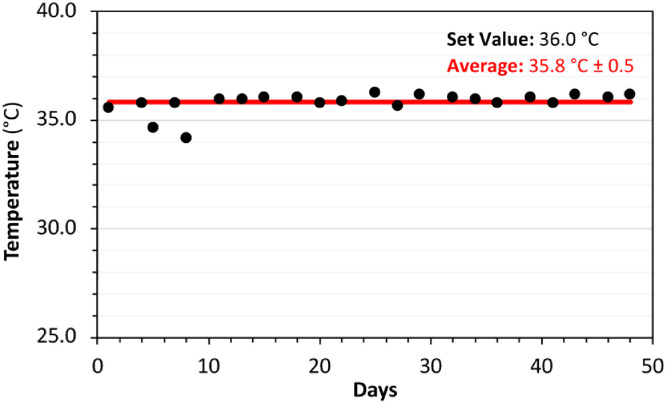
Temperature variation of the bioreactor during a long-test.

Maintaining stable temperature conditions are often required by the anaerobic microorganisms. Mostly mesophilic conditions are in practice for many cases. In this developed packed-bed bioreactor system, it was also aimed to keep the temperature around 34 – 38 °C which is a widely used level for anaerobic MMC. In Fig. 8a long-term temperature monitoring graphic of one tetrapod bioreactor during an anerobic test, is presented. Temperature was set at 36 °C by the temperature control system. Results showed a satisfactory stable temperature environment with a low standard deviation (± 0.5 °C). During a 50 days of continuous fermentation experiment, average temperature of bioliquid was estimated as 35.8 °C.

The packed-bed was able to distribute temperature, maintaining a stable liquid temperature during all experimental operation time. It must be noticed that since the temperature is applied from the outside of the glass-bottle reactor, it's important to maintain a constant liquid recirculation through the packed-bed, which avoids overheating of certain places that can damage the microbial biofilm.

### Gas leaking tests

**Fig. 9:**
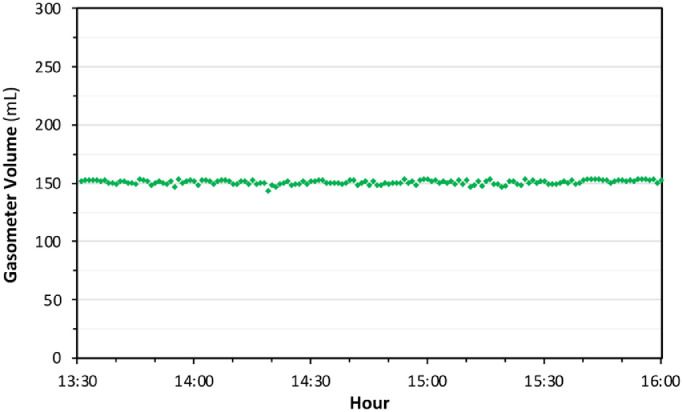
Hydrogen (H_2_) leaking test.

Before fermentation tests, bioreactor system was tested to assess impermeability of the assembled tetrapod. Reactor and gasbag were filled with a known amount of hydrogen (H_2_) gas as a most permeable gas. H_2_ leaking test lasted two and a half hours. During the H_2_ test, the digital gasometer has shown a stable gas amount (Fig. 9) and concentration change of gases were in negligible level at the end.

**Fig. 10:**
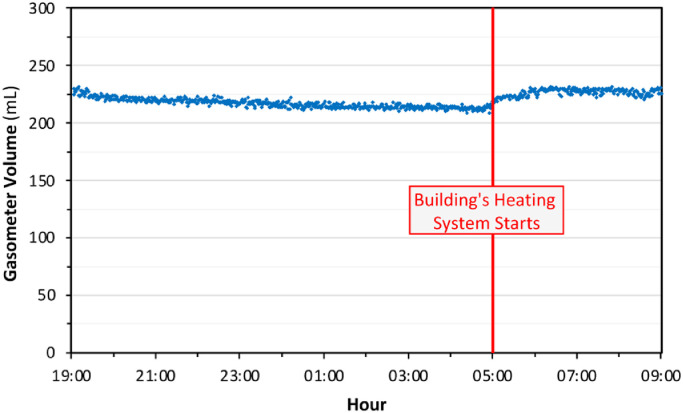
Gas leaking and monitoring test under simulated operational conditions (Italy, December 2020).

Another gas leaking test was conducted overnight using nitrogen as a filling gas. In this time, the tetrapod bioreactor was filled with a known amount of water. The internal temperature of the reactor was set to 36 °C and water recirculation was initiated, to simulate an on-going anaerobic test. During this second gas leaking test, digital gasometer and impermeability of the reactor system has shown a good performance once again (Fig. 10). The test was conducted during a winter night, so that the gasometer, which is not included the temperature heating system of the tetrapod set-up, was affected by the surrounding temperature conditions. The slight negative slope between the beginning time until 05:00 AM when the building's heating starts, and the following sharp increase in total gas amount, were correlated with this fact. However, the initial and final total gas volumes were quite similar and consistent. This trend obtained by the digital gasometer is also again verifying the consistency of the gas-monitoring system.

### Mass balance in terms of COD

COD as a commonly used direct measurement method for the monitoring of organic substances, can serve as a platform unit for overall evaluation of this kind of complex bioreactor operations. Since it basically represents the amount of oxygen needed for the complete oxidation of total organic matter in a given volume, it is a very useful indirect measurement tool for the measurement of chemical energy in both liquid, solid and gaseous materials. For this reason, COD was used as a single-unit to evaluate overall performance of tetrapod bioreactor system during a 10-days of anaerobic fermentation test. All the operational methods were identical for both bioreactors. During the continuous operation test, glucose was the sole substrate for the already enriched anaerobic MMC which was initially originated from a commercial anaerobic digester. Daily feeding and discharging cycles were applied with 20 g-COD/L input concentration. All input and output liquid samples were measured with a Quick COD analyzer (LAR Process Analyzer AG) by following the ASTM D6238–98 method. The output gas samples were measured by a Gas Chromatography (GC) system equipped with a Thermal Capture Detector (TCD) [22]. Results of the TCD analysis were manually calculated as COD by using the daily gas volumes via digital gasometer. Volatile fatty acids in liquid samples were also monitored by a GC mass spectrometer (MS) equipped with DB-FFAP polar column, for the evaluation of acid conversion efficiency [23].

**Fig. 11:**
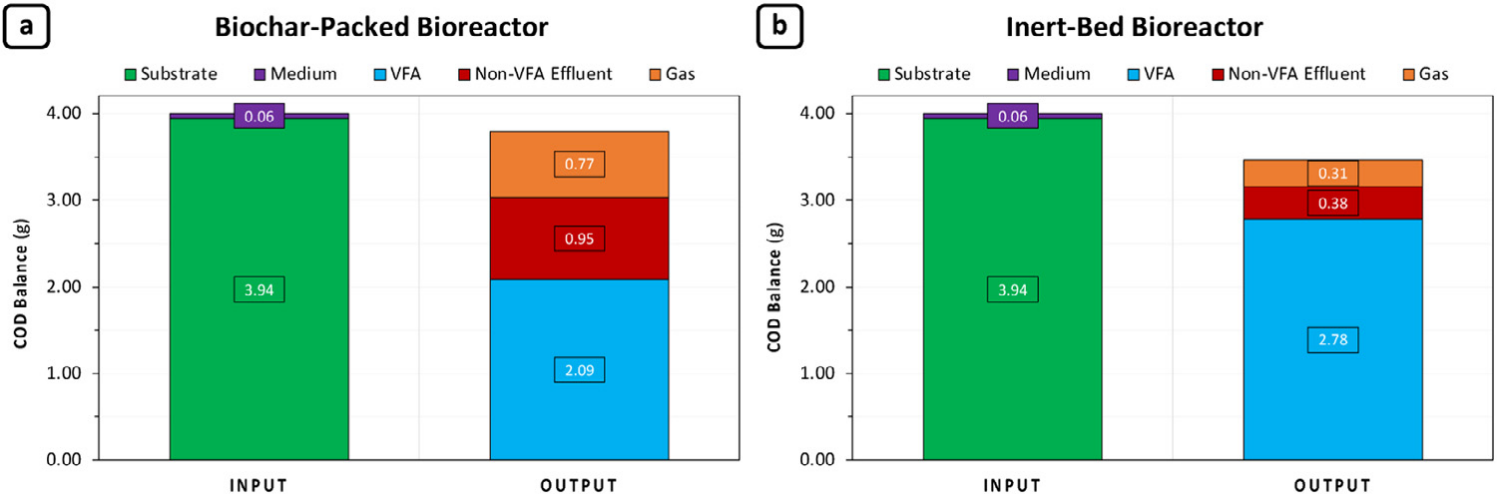
Overall COD balance of a 10-day anaerobic test which was conducted in tetrapod system.

Overall COD balance of the conducted anaerobic test is presented in below graphs, which reveals a good performance for both tetrapod bioreactor systems; one is with biochar (Fig. 11a), the second is with an inert-bed (Fig. 11b). The anaerobic test conducted in biochar-packed tetrapod was resulted in very satisfying COD closure (95%) and once again proved the efficiency of the developed bioreactor system. While inert-bed bioreactor was shown a relatively lower COD closure with 80%, it may be linked with a continuous biofilm formation which was not included in COD balance. A possible reason behind this difference can be correlated with a potentially more robust and consistent biofilm formation already occurred at previous enrichment-phase in porous biochar filter media, as compared to inert spherical glassbeads material. For this reason, daily glucose feeding was more likely to be used a bit more for regeneration of MMC biofilm in inert-bed bioreactor as compared to biochar-packed one. However, a higher VFA output (in terms of COD) was observed in inert-bed with a lower gas output COD. This result can be correlated with a possible methanogenic toxification resulted from VFA accumulation in the inert-bed tetrapod. While in biochar-packed bioreactor this phenomenon possibly mitigated by biochar itself. So that, a higher amount of biogas production in COD basis was expected to be occurred, as it happened (Fig. 11a).

### Further improvements

The developed ‘tetrapod’ anaerobic bioreactor was proven as a valid methodologic system for lab-scale anaerobic tests, by this Method Article. This novel bioreactor system which equipped with; Arduino based electronic controlling system, an original digital gasometer with 3D-Printed biodegradable (PLA) holding structure, and easy-to-operate sampling ports, can be considered as a useful tool for most of the wet-type anaerobic experimental studies. Given the flexible Arduino platform and availability of dozens of different sensors, the system can be improved to expand the bioreactor's monitoring and control capabilities.

## Appendix Supplementary materials

Image, application 1
